# Synergy of 1,25-dihydroxyvitamin D3 and carboplatin in growth suppression of SKOV-3 cells

**DOI:** 10.3892/ol.2014.2307

**Published:** 2014-07-02

**Authors:** ZENGLI ZHANG, HEMEI ZHANG, ZHIYONG HU, PING WANG, JIANMEI WAN, BINGYAN LI

**Affiliations:** 1 Department of Toxicology, School of Public Health, Soochow University, Suzhou, Jiangsu 215123, P.R. China; 2 Li Shui Center for Disease Control and Prevention, Lishui, Zhejiang 323000, P.R. China

**Keywords:** 1,25-dihydroxyvitamin D3, carboplatin, ovarian cancer, SKOV-3 cells

## Abstract

1α,25-Dihydroxyvitamin D3 [1,25(OH)_2_D_3_] has been demonstrated to inhibit the growth of cancer cells. However, carboplatin is the most widely used chemotherapeutic agent to treat cancer. We hypothesized that vitamin D may enhance the antiproliferative effects of carboplatin, and tested this hypothesis in ovarian cancer SKOV-3 cells treated with carboplatin and 1,25(OH)_2_D_3_. Cell viability was determined by Cell Counting Kit-8, while cell cycle distribution, apoptosis, reactive oxygen species (ROS) and mitochondrial membrane potential (MMP) were analyzed by flow cytometry. In these experiments, 1,25(OH)_2_D_3_ and carboplatin each provided dose-dependent suppression of SKOV-3 growth, and synergy was demonstrated between 10 nM 1,25(OH)_2_D_3_ and carboplatin. The proportion of cells in G_0_/G_1_ phase was markedly reduced by the drug combination, while the proportion of cells in G_2_/M phase was increased. Apoptosis did not increase in ovarian cancer cells treated with 10 nM 1,25(OH)_2_D_3_ alone; however, 1,25(OH)_2_D_3_ evidently enhanced carboplatin-induced apoptosis. Similarly, ROS production was evidently higher and MMP was lower in cells treated with the two drugs than in those treated with each drug alone. The results suggested that 1,25(OH)_2_D_3_ suppresses SKOV-3 growth and enhances the antiproliferative effect of carboplatin. The drugs function synergistically by inducing cell cycle arrest, increasing apoptosis and ROS production, and reducing MMP.

## Introduction

Ovarian cancer is a serious global threat to female health and is a leading cause of cancer-related mortality in females, often due to late-stage recognition and aggressive tumor relapse (1). High patient morbidity is attributable in part to the recurrent growth of residual ovarian tumor cells that become resistant to standard chemotherapeutic treatments, and then aggressively proliferate and spread or metastasize to multiple sites. In total, 70% of females diagnosed with ovarian cancer present with advanced malignant disease and usually undergo surgery followed by a combination of paclitaxel and platinum-based chemotherapy (2,3). However, recurrences occur in the majority of patients, and only ~30% of patients with distant metastases survive five years following diagnosis (4,5). Failure of chemotherapy in recurrent ovarian cancer is usually due to the development of resistance to one or the two main classes of chemotherapy agents used to treat ovarian cancer (6–8). Novel therapeutic approaches are therefore necessary for the management of advanced and recurrent ovarian cancer.

Platinating agents, such as carboplatin, are potent chemotherapeutic agents widely used for the adjuvant treatment of primary ovarian cancer and metastatic disease. The drug induces the formation of DNA adducts, G_2_ phase cell cycle arrest and the subsequent triggering of apoptosis. However, the efficacy of carboplatin is limited by drug resistance and side-effects, including nephrotoxicity, myelosuppression and neurotoxicity (9,10). The mechanisms underlying the development of resistance to platinating agents, particularly carboplatin, include the repair of DNA lesions, translesion DNA synthesis, altered drug transport, increased antioxidant production and reduction of apoptosis (11–13). Altered gene expression affecting cellular transport, DNA repair, apoptosis and cell-cell adhesion are the mechanisms of platinum resistance that have been observed in patient samples (14,15). Therapeutic success may therefore be improved if tumor cells can be sensitized to carboplatin treatment with a combination therapy.

1α,25-Dihydroxyvitamin D3 [1,25(OH)_2_D_3_] is the most active metabolite of vitamin D3. It is a scarce natural product that is synthesized predominantly in the skin from 7-dehydrocholesterol by exposure to ultraviolet sunlight. Although its classical role as the major regulator of calcium homeostasis and bone formation/resorption has been recognized for some time (16), more recent findings suggest that 1,25(OH)_2_D_3_ is an important modulator of cellular proliferation and differentiation in a variety of benign and malignant cells. 1,25(OH)_2_D_3_ also exhibits anti-invasion, antiangiogenesis and antimetastatic activity *in vivo* (17–21) and acts as a chemopreventive agent in animal models of lung, colorectal and breast cancer (22–24).

The aim of this study was to determine whether 1,25(OH)_2_D_3_ enhances the cytostatic effects of carboplatin in SKOV-3 cells and to characterize the mechanism of its effect.

## Materials and methods

### Cell culture and agents

The human ovarian serous papillary cystadenocarcinoma SKOV-3 cell line was purchased from the Type Culture Collection of the Chinese Academy of Sciences (TCCCAS; Shanghai, China) and was verified as mycoplasma free. Authenticity of the cell line was confirmed by the TCCCAS. The SKOV-3 cells were maintained in RPMI 1640 medium supplemented with 10% fetal bovine serum, 100 U/ml penicillin and 5 mg/ml streptomycin. These agents and trypsin-EDTA solution were purchased from Invitrogen Life Technologies (Carlsbad, CA, USA). 1,25(OH)_2_D_3_ and carboplatin were purchased from Sigma-Aldrich (St. Louis, MO, USA) and Qilu Pharmaceutical Co., Ltd. (Jinan, China), respectively. 1,25(OH)_2_D_3_ was dissolved in ethanol and stored in a concentrated solution (10^−5^ mol/l) at −80°C. The 1,25(OH)_2_D_3_ was freshly diluted in RPMI 1640 prior to each experiment. The ethanol concentrations in each experiment were ≤0.1%. The carboplatin solution was prepared with sterile distilled water and fresh stocks were prepared on the day of each experiment, and dilutions were prepared with RPMI 1640.

### Cell viability assay

The viability of SKOV-3 cells was determined by Cell Counting Kit-8 (CCK-8; Dojindo Laboratories, Kumamoto, Japan). Briefly, cells at the exponential phase were collected, transferred to 96-well plates (2,000 cells/well) and cultured overnight. The plating medium was removed and replaced with a medium containing the appropriate concentration of vehicle (0.1% ethanol), 1,25(OH)_2_D_3_ (0.1, 1, 5, 10, 50, 100, 200 and 500 nM) or carboplatin (0.2, 2, 20, 40, 80 and 160 mg/l). The combined effects were evaluated by incubation with 1,25(OH)_2_D_3_ and carboplatin. Cells were allowed to grow for an additional 72 h, then 10 μl of CCK-8 solution was added and the cells were incubated for 1 h. Absorbance (Abs) was measured at 450 nm in a microplate reader (BioTec Instruments, Inc., Winooski, VT, USA) and growth inhibition was calculated as the percentage difference of the treated cells versus the vehicle controls, according to the following formula: Inhibition rate (%) = [(Abs of vehicle control cells - Abs of treated cells)/Abs of vehicle control cells] × 100. Each experiment was performed in triplicate.

Data were analyzed using KaleidaGraph (Synergy Software, Reading, PA, USA) to determine the drug IC_50_ value. The combined index (CI) was used to evaluate the drug combination assays according to the following formula (25): CI = D_A_/IC_50,A_ + D_B_/IC_50,B_, where D_A_ is the IC_50_ of drug A when A was combined with B, D_B_ is the IC_50_ of drug B when A was combined with B, IC_50,A_ is the IC_50_ of drug A, and IC_50,B_ is the IC_50_ of drug B. Each CI was calculated from the mean affected fraction at each drug ratio concentration in triplicate. CI>1, CI=1, and CI<1 indicated antagonism, additive effect or synergy, respectively (26).

### Cell cycle analysis

SKOV-3 cells were grown to 50% confluence in 35-mm dishes and treated with the vehicle control, 10 nM 1,25(OH)_2_D_3_, 40 mg/l carboplatin, or a combination of the two drugs for 72 h. The cells were harvested by pooling the floating cells with the trypsinized monolayers and were pelleted by centrifugation at 179 × g for 5 min. Following fixation with cold 75% ethanol, the cells were resuspended in a solution of phosphate-buffered saline (PBS; pH 7.4) containing 25 mg/ml propidium iodide (PI; Sigma-Aldrich), 0.1 mM EDTA (Invitrogen Life Technologies) and 0.01 mg/ml DNase-free RNase (Invitrogen Molecular Probes, Inc., Eugene, OR, USA). The samples were incubated for 15 min at room temperature prior to cell cycle analysis using a FC500 flow cytometer (Beckman Coulter, Fullerton, CA, USA). Statistics were performed on 20,000 events per sample using MultiCycle DNA Content and DNA cell cycle analysis software (MutiCycle AV for Windows; Phoenix Flow System, Inc., San Diego, CA, USA).

### Apoptosis assay

The number of apoptotic cells was determined using the Alexa Fluor 488 Annexin V/Dead Cell apoptosis kit (Invitrogen Life Technologies). Following treatment, the cells were harvested and washed with PBS, then suspended in PBS with PI and Annexin V. The cell suspensions were incubated in the dark for 15 min at 37°C and then analyzed on a FC500 flow cytometer.

Confocal laser-scanning microscopy was performed using an SP-2 confocal laser-scanning microscope (Leica, Wetzlar, Germany) equipped with an oil immersion objective (63X). Nuclear images were obtained at an excitation wavelength of 405 nm of 4′,6-diamidino-2-phenylindole (DAPI).

### Measurement of mitochondrial membrane potential (MMP)

MMP was measured using JC-1 dye (Invitrogen Life Technologies), a cationic dye that aggregates in the mitochondria of healthy cells; at high concentrations, JC-1 monomers (green fluorescence) form aggregates (red fluorescence). The ratio of the green/red fluorescence is independent of mitochondrial shape, density or size, and depends only on the membrane potential. MMP analysis was performed as previously described (27). Briefly, SKOV-3 cells were treated for 72 h, then harvested and stained with 10 μM JC-1 at 4°C for 1 h prior to flow cytometry analysis. JC-1 was excited with the 488-nm argon laser (Beckman Coulter) and JC-1 green and red fluorescence was recorded using 530 nm ± 15 nm and a 590 nm ± 15 nm band pass filter channels. A minimum of 20,000 cells within the gated region were analyzed. The cell sorting gates used were FL-2 versus FL-1 blotting (28). The ratio of the fluorescence intensity at 590 nm to that at 530 nm (FL-2:FL-1 ratios) was considered the relative MMP value. Data are presented as the mean of three experiments.

### Measurement of intracellular reactive oxygen species (ROS)

Intracellular ROS was measured by a cell-permeating probe, 5-[and-6]-chloromethyl-2′,7′-dichlorodihydrofluorescein diacetate, acetyl ester (CM-H_2_DCFDA, Invitrogen Molecular Probes), as previously described (29). CM-H_2_DCFDA is a non-polar compound that is hydrolyzed upon cell entry, forming a non-fluorescent derivative that can be converted into a fluorescent product in the presence of a true oxidant. Mean fluorescence intensity was used as measure of ROS level as determined by flow cytometer. Cells were treated for 72 h, washed and loaded with 10 μM CM-H_2_DCFDA for 1 h. Green fluorescence intensity was used as a measure of relative intracellular ROS by flow cytometry at 530 nm. A total of 20,000 cells within the gated region were analyzed. Data are presented as the mean of three experiments

### Statistical analysis

Statistical analysis was performed using SPSS, version 13.0 for Windows (SPSS, Inc., Chicago, IL, USA). Data are presented as the mean ± standard deviation. One-way analysis of variance was used to evaluate differences between the groups. P<0.05 was considered to indicate a statistically significant difference.

## Results

### Dose-response of SKOV-3 cells to 1,25(OH)_2_D_3_ and carboplatin

**Figure 1:**
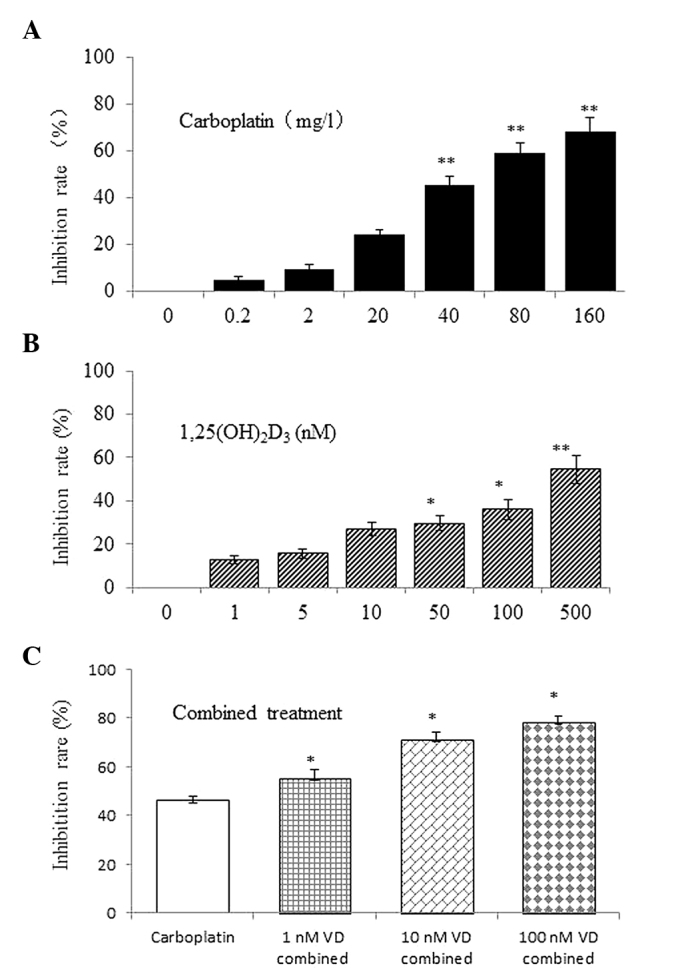
1,25(OH)_2_D_3_ and/or carboplatin inhibition of SKOV-3 cell growth. Growth inhibition increased with increasing doses of (A) carboplatin and (B) 1,25(OH)_2_D_3_ over 72 h. ^*^P<0.05 and ^**^P<0.01, vs. the vehicle control. (C) Growth inhibition also increased with combined 1,25(OH)_2_D_3_ and carboplatin treatment. ^*^P<0.05, vs. single-drug treatment. 1,25(OH)_2_D_3_, 1,25-dihydroxyvitamin D_3_.

Initial experiments were performed to determine the range of drug concentrations that would elicit growth inhibition in ovarian cancer cells. SKOV-3 cells were incubated with graded 1,25(OH)_2_D_3_ (0.1, 1, 5, 10, 50, 100, 200 and 500 nM) and carboplatin (0.2, 2, 20, 40, 80, and 160 mg/l). In the cell viability assay, 1,25(OH)_2_D_3_ inhibited growth in a dose-dependent manner (Fig. 1A). Carboplatin also suppressed the viability of SKOV-3 cells in a similar manner (Fig. 1B). The differences between the vehicle control and test groups were statistically significant (P<0.05). Based on 10–90% inhibition rates of cell growth in the KaleidaGraph program, the IC_50_ values of 1,25(OH)_2_D_3_ and carboplatin were 420.45 nM (R^2^=0.9904) and 54.6 mg/l (R^2^=0.9923), respectively.

### Combined administration of 1,25(OH)_2_D_3_ and carboplatin

To determine whether the drugs work synergistically, SKOV-3 cells were treated with carboplatin in the presence of 1,25(OH)_2_D_3_. The growth inhibition was significantly greater with the combined treatment (Fig. 1C) and greater synergy was achieved at 40 mg/l carboplatin in combination with 10 nM 1,25(OH)_2_D_3_ (CI=0.57). The IC_50_ of carboplatin evidently decreased with increasing 1,25(OH)_2_D_3_ concentrations (Table I).

### Cell cycle analysis

**Figure 2:**
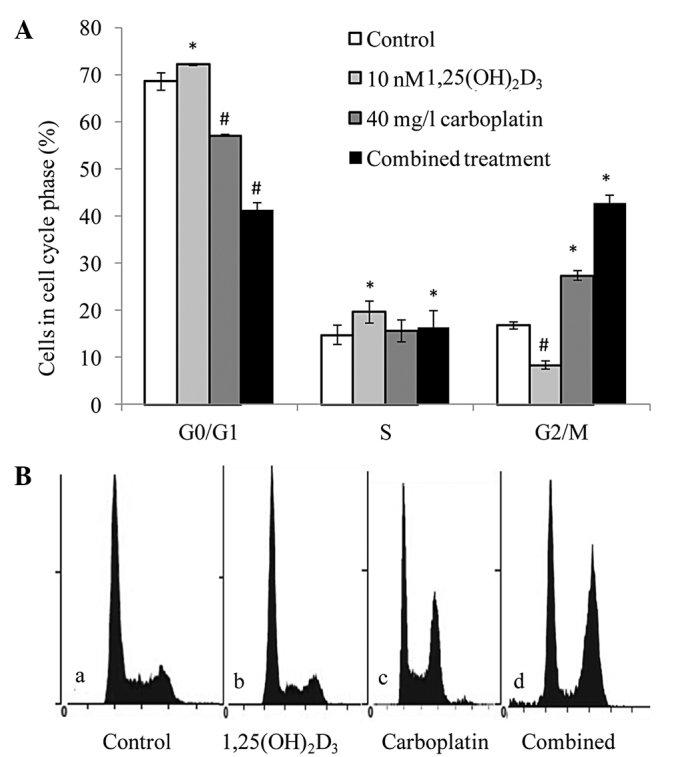
Combined effect of 1,25(OH)_2_D_3_ and carboplatin on cell cycle distribution. (A) G_2_/M cell cycle arrest in SKOV-3 cells treated with 1,25(OH)_2_D_3_ and carboplatin versus the untreated cells. ^*/#^P<0.05, vs. the vehicle controls. (B) Flow cytometric analysis: a, control group; b, 10-nM 1,25(OH)_2_D_3_ group; c, 40-mg/l carboplatin group; and d, combined treatment group. Similar results were obtained in all three experiments. 1,25(OH)_2_D_3_, 1,25-dihydroxyvitamin D_3_.

To determine whether 1,25(OH)_2_D_3_ enhancement of the antiproliferative activity of carboplatin was due to alterations in the cell cycle, the cell cycle distribution of SKOV-3 cells treated with the vehicle control, 10 nM 1,25(OH)_2_D_3_, 40 mg/l of carboplatin and the drugs in combination were compared. Compared with the vehicle control, 1,25(OH)_2_D_3_ significantly increased the percentage of cells in G_0_/G_1_ phase, accompanied by a reduction of cells in G_2_/M phase. The reverse effect occurred with carboplatin; a decrease in cells in G_0_/G_1_ phase and an increase in cells in G_2_/M phase was observed. However, the percentage of cells in S phase changed very little. Following treatment with 10 nM 1,25(OH)_2_D_3_ and 40 mg/l carboplatin, the distribution of G_0_/G_1_-phase cells in SKOV-3 cells was further reduced, while cells in G_2_/M phase evidently increased (Fig. 2A and B). Therefore, it was concluded that the combined treatment had a similar effect as carboplatin treatment alone, but this observation regarding cell cycle arrest requires more consideration.

### Apoptosis in combination treatment of 1,25(OH)_2_D_3_ and carboplatin

**Figure 3:**
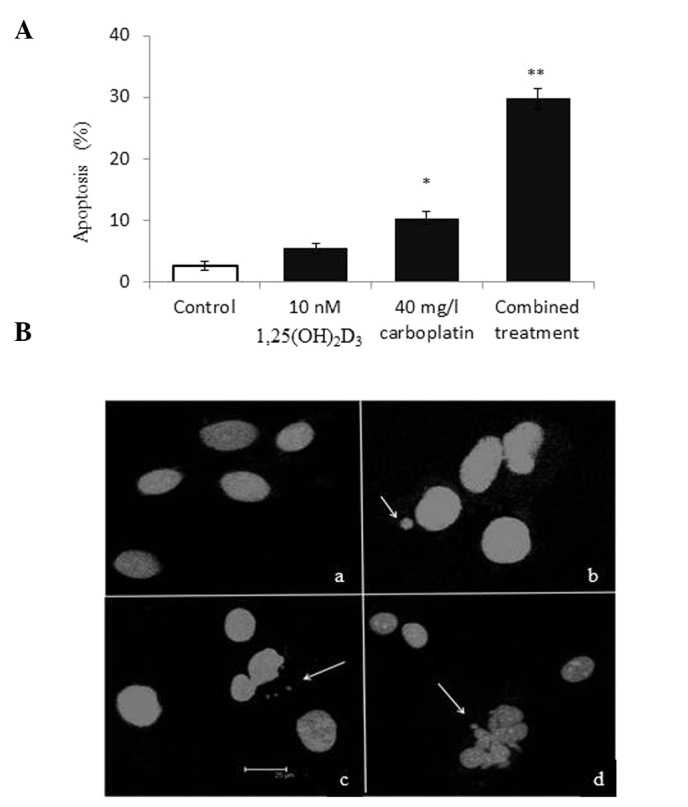
Combined effect of 1,25(OH)_2_D_3_ and carboplatin on apoptosis in SKOV-3 cells. (A) Apoptosis increased following the combined treatment of 1,25(OH)_2_D_3_ and carboplatin versus the untreated cells. ^*^P<0.05 and ^**^P<0.01, vs. the vehicle controls. (B) Nuclei of SKOV-3 cells (stain, 4′6-diamidino-2-phenylindole): a, control group; b, 10-nM 1,25(OH)_2_D_3_ group; c, 40-mg/l carboplatin group; and d, combined treatment group. Arrows indicate the fragmented DNA from the nuclei. 1,25(OH)_2_D_3_, 1,25-dihydroxyvitamin D_3_.

Apoptosis was assessed to identify the mechanism of growth inhibition by the combined treatment of 1,25(OH)_2_D_3_ and carboplatin in ovarian cancer cells. Apoptosis was increased in SKOV-3 cells treated with 40 mg/l carboplatin, but the increase was not significant in cells treated with 10 nM 1,25(OH)_2_D_3_ (a dose of 100 nM did increase apoptosis, data not shown) compared with the control group. However, apoptosis was evidently increased by combined treatment (Fig. 3A). Furthermore, confocal laser-scanning microscopy with DAPI staining demonstrated that cells treated with the two drugs exhibited apoptotic nuclei with DNA fragmentation, chromatin condensation and formation of apoptotic bodies (Fig. 3B), and that cells treated with single drugs contained fewer apoptotic nuclei than cells treated with both drugs.

### MMP change following combination treatment

**Figure 4:**
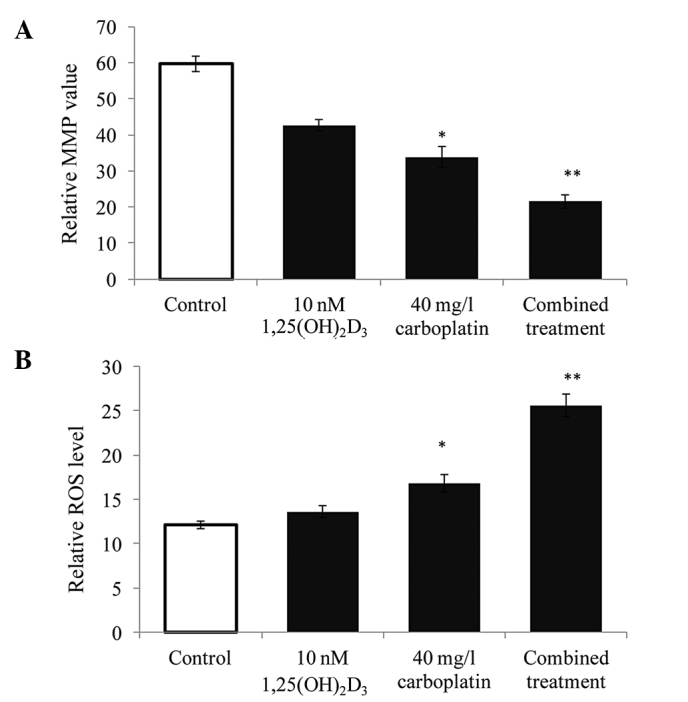
Combined effect of 1,25(OH)_2_D_3_ and carboplatin on ROS production and MMP loss in SKOV-3 cells. (A) MMP was reduced in SKOV-3 cells following combination treatment versus the controls. (B) ROS production increased in SKOV-3 cells following the combined treatment versus the untreated controls. ^*^P<0.05 and ^**^P<0.01, vs. the vehicle controls. 1,25(OH)2D3, 1,25-dihydroxyvitamin D3; ROS, reactive oxygen species; MMP, mitochondrial membrane potential.

Depolarization of MMP is a characteristic feature of apoptosis; hence, treated cells were examined for a drop in MMP. MMP was not found to significantly reduce with 10 nM of 1,25(OH)_2_D_3_ in SKOV-3 cells, but MMP dropped in cells treated with 40 mg/l carboplatin. The maximal effect was obtained with combined 1,25(OH)_2_D_3_ and carboplatin treatment (Fig. 4A). Each drug alone inhibited growth, increased apoptosis and reduced MMP. Thus, 1,25(OH)_2_D_3_ further reduces the MMP of ovarian cells induced by carboplatin; however, 10 nM of 1,25(OH)_2_D_3_ alone did not reduce MMP_._

### ROS production following combined treatment

Oxidative stress appears to be critical for tumor therapy, as ROS overproduction corresponds to an increase in apoptosis. In order to assess whether the growth suppression of 1,25(OH)_2_D_3_ and carboplatin is due to ROS production, ROS were measured as detected by CM-H_2_DCFDA and expressed as the mean fluorescence by flow cytometry in comparison with the vehicle controls. The ROS levels in cells treated with 40 mg/l carboplatin were evidently increased, while those of cells treated with 10 nm 1,25(OH)_2_D_3_ were not found to significantly increase in comparison with the vehicle control. Although carboplatin treatment alone increases ROS production, the combined treatment produced a clear increase in ROS levels compared with the vehicle control (Fig. 4B). Thus, the growth inhibition of ovarian cancer cells can be induced by the increase of ROS triggered by the combined treatment.

## Discussion

The primary actions of 1,25(OH)_2_D_3_ are mediated through the nuclear vitamin D receptor (VDR), a member of the steroid/thyroid hormone superfamily of ligand-activated transcription factors. VDR has been found in rat ovaries by immunohistochemistry (30), as well as hen ovaries by ligand binding assays (31), indicating that the ovary is a target organ for vitamin D. Studies have also shown that VDR is expressed in gynecologic neoplasms, such as ovarian cancer (32,33), which suggests that 1,25(OH)_2_D_3_ may be effective against ovarian cancer. The correlation between vitamin D and the risk of ovarian cancer has also received unprecedented attention. Several ecological studies have reported that ovarian cancer mortality inversely correlates with sun exposure, which initiates vitamin D production in the skin (34,35). Other studies of dietary intake of vitamin D have also observed an inverse correlation with ovarian cancer risk (36,37). Therefore, the inverse correlation between vitamin D level and ovarian cancer-related mortality suggests that the insufficiency of 1,25(OH)_2_D_3_ may contribute to ovarian cancer initiation and/or progression.

In this study, 1,25(OH)_2_D_3_ was demonstrated to inhibit ovarian cancer SKOV-3 cell growth in a dose-dependent manner. It also markedly enhanced the inhibitory effects of carboplatin on ovarian cancer cells at a concentration of 10 nM 1,25(OH)_2_D_3_. While this has been viewed as the major anticancer effect for 1,25(OH)_2_D_3_, its mechanism remains uncertain.

Cell-cycle perturbation is central to chemotherapy-mediated antiproliferative activity in tumor cells, and combined treatment with 1,25(OH)_2_D_3_ and carboplatin in the current study led to a significant increase in the percentage of G_2_/M-phase cells and an evident decrease in G_0_/G_1_-phase cells. Moffatt *et al* (38) also demonstrated that, over time, combined 1,25(OH)_2_D_3_ and carboplatin increases the percentage of prostate cancer cells in G_2_/M phase. The authors found this trend to correlate with an apparent decrease in the amount of cells in G_1_ phase. Studies of ovarian cells have suggested that 1,25(OH)_2_D_3_ causes cell cycle arrest at the G_1_/S and G_2_/M checkpoints. In addition, these studies have shown that the proliferation of ovarian cancer OVCAR3 cells is suppressed by 1,25(OH)_2_D_3_ (33) and that 1,25(OH)_2_D_3_ arrests ovarian cancer cells in G_2_/M phase by a mechanism that involves GADD45 (39). They have also indicated that 1,25(OH)_2_D_3_ arrests ovarian cancer cells in the G_1_ phase by increasing the abundance of p27, an inhibitor of cyclin-dependent kinase activity (40). 1,25(OH)_2_D_3_ enhanced the effects of platinum agents by inhibiting the growth of the breast cancer cell line, MCF-7 (41). Additionally, *in vivo* evidence for the positive interaction between 1,25(OH)_2_D_3_ and platinum compounds has been obtained in a murine squamous cell carcinoma model system (42). These studies have shown that 1,25(OH)_2_D_3_ causes cell cycle arrest and growth suppression in ovarian cancer cells.

The results of the current study indicated that 1,25(OH)_2_D_3_ enhances the growth-inhibitory effect of carboplatin by increasing the rate of apoptosis. Furthermore, 1,25(OH)_2_D_3_ induced apoptosis, possibly due to increased ROS production, which directly induces single- and double-strand breaks, abasic sites and DNA fragmentation, all of which lead to apoptosis. Koren *et al* (43) reported that 1,25(OH)_2_D_3_ induces ROS production in MCF-7 cells and suggested a compensatory mechanism in which growth arrest is induced by oxidative stress, while antioxidant activities are increased. 1,25(OH)_2_D_3_ was not found to act synergistically with anticancer cytokines in the tumor milieu, which is mediated by ROS (44). In the present study, 10 nM 1,25(OH)_2_D_3_ was not found to induce ROS production alone, but to enhance ROS production in ovarian cancer cells treated with carboplatin. These studies have demonstrated that the anticancer activity of 1,25(OH)_2_D_3_ is associated with the pro-oxidant action of 1,25(OH)_2_D_3_ its in MCF-7 cells, which may be the result of increased intracellular ROS. However, overproduction of ROS through endogenous or exogenous sources may induce DNA damage, the accumulation of which may lead to multistep carcinogenesis (45). Thus, the antioxidative effects of vitamin D have been suggested by epidemiological surveys and numerous *in vitro* and *in vivo* laboratory studies (46,47). The antioxidative effect of vitamin D strengthens its roles in cancer chemoprevention and adds to a growing list of the beneficial effects of vitamin D in cancer (48).

One important observation from this study is that 1,25(OH)_2_D_3_ enhances the carboplatin-induced apoptosis of ovarian cells and is associated with the loss of MMP. Chen *et al* (49) also demonstrated that ergocalciferol, vitamin D2, causes HL-60 cell apoptosis via a drop in MMP. 1,25(OH)_2_D_3_ has also been found to augment the loss of MMP induced by TNF (50). Another finding has suggested that 1,25(OH)_2_D_3_ sensitizes breast cancer cells to ROS-induced death by influencing the caspase-dependent and -independent modes of cell death, upstream of mitochondrial damage (51). Therefore, 1,25(OH)_2_D_3_ enhances the anticancer effects of carboplatin through production of ROS and loss of MMP. Other studies have found that 1,25(OH)_2_D_3_ induces ovarian cancer cell apoptosis by downregulating telomerase, thus modulating telomere integrity or perhaps via a telomerase-independent mechanism (52). This result also indicates that 1,25(OH)_2_D_3_ may induce ovarian cancer cell apoptosis through various mechanisms.

In conclusion, the present study demonstrated that 1,25(OH)_2_D_3_ is a potent inhibitor of ovarian cancer cell growth *in vitro*, and enhances the therapeutic effects of carboplatin by altering the cell cycle and increasing apoptosis through changes in ROS and MMP. These findings suggest the potential utility of combining 1,25(OH)_2_D_3_ with cytotoxic agents for the treatment of ovarian cancer.

## Figures and Tables

**Table I:** Effect of 1,25(OH)_2_D_3_ and carboplatin combination on the growth of SKOV-3 cells.

1,25(OH)_2_D_3_, nM	IC_50_^a^ of carboplatin, mg/l	CI^b^
0	54.6	NA^c^
1	35.0	0.78
10	26.7	0.57
100	24.4	0.76

a IC_50_ (half the inhibitory concentration) of 1,25(OH)_2_D_3_ was 420.45 nM.

b CI>1, antagonism; CI=1, additive effect; and CI<1, synergy.

c It was not possible to calculate the CI with a concentration of 0 nm 1,25(OH)_2_D_3_.

CI, combined index; 1,25(OH)_2_D_3_, 1,25-dihydroxyvitamin D_3_.
